# P-507. Do we understand congenital syphilis surveillance in this country? How to improve surveillance of congenital syphilis — Japan, 2024

**DOI:** 10.1093/ofid/ofaf695.722

**Published:** 2026-01-11

**Authors:** Mizue Eita, Takuya Yamagishi, Hirofumi Kato, Tomoe Shimada, Ayu Kasamatsu, Takuri Takahashi, Taro Kamigaki, Motoi Suzuki, Yukihiro Akeda, Tomimasa Sunagawa

**Affiliations:** National Institute of Infectious Diseases/ Kyoto Prefectural Government, chiyoda-ku, Tokyo, Japan; Japan Institute for Health Security, Shinjuku, Tokyo, Japan; National Institute of Infectious Diseases, Shinjuku-ku, Tokyo, Japan; National Institute of Infectious Deaseases, chiyoda-ku, Tokyo, Japan; Japan Institute for Health Security, Shinjuku, Tokyo, Japan; National Institute of Infectious Diseases, Shinjuku-ku, Tokyo, Japan; Japan Institute for Health Security, Shinjuku, Tokyo, Japan; Japan Institute for Health Security, Shinjuku, Tokyo, Japan; National Institute of Infectious Diseases, Shinjuku-ku, Tokyo, Japan; National Institute of Infectious Diseases, Shinjuku-ku, Tokyo, Japan

## Abstract

**Background:**

Reflecting a nationwide heterosexual outbreak of syphilis, the number of congenital syphilis (CS) notifications have been increasing in Japan, reaching 37 notifications in 2023, approximately 1.5 times that in 2022. The reporting criteria for CS require either meeting specific laboratory criteria (e.g., anti-cardiolipin antibody [aCL] titer elevated persistently beyond 6 months of age) or exhibiting symptoms compatible with CS. To know to what extent current surveillance provides the necessary information to assess progress of both national and global strategies, we evaluated the national surveillance of CS. Our evaluation purposes were to assess how well the surveillance captures the true cases of CS and collects the information useful for CS prevention, such as mothers’ information.

**Methods:**

We used the surveillance system evaluation method published by the US Center for Disease Control and Prevention. An extracted national surveillance data between January 2021 and June 2024 was used for quantitative assessment. We then interviewed officials in local government, local public health centers, and physicians in hospitals in 2024.

**Results:**

Ninety-one CS cases are notified during the study period. Physicians noted in the interview that there is a possibility to miss cases because it is difficult to check persistent high aCL titers in asymptomatic neonates. Information on testing details were not provided for 13 of 45 cases (29%) that were reported to test positive for IgM tests. This means overreporting could not be completely ruled out. No maternal information was included in 11 notifications (12%), and even for those with maternal information, only 27 notifications (30%) mentioned the treatment history of maternal syphilis. Doctors noted it is difficult to obtain a sensitive sexual history in the role as a pediatrician although the sexual history of the mothers is critical for building a prevention strategy.

**Conclusion:**

The CS notification criteria do not fully capture the true number of cases, and modification of the criteria should be considered from clinical and public health perspectives. Additionally, the information useful for the prevention of CS was only partially collected under the current surveillance, and we need to revisit necessary items that meet prevention needs.

**Disclosures:**

All Authors: No reported disclosures

